# Wearable Cardioverter Defibrillator Shortens the Lengths of Stay in Patients with Left Ventricular Dysfunction after Myocardial Infarction: A Single-Centre Real-World Experience

**DOI:** 10.3390/jcm12154884

**Published:** 2023-07-25

**Authors:** Laura Sofia Cardelli, Quentin Delbaere, François Massin, Mathieu Granier, Gianni Casella, Gaetano Barbato, Valentin Dupasquier, Jean-Christophe Macia, Florence Leclercq, Jean-Luc Pasquie, François Roubille

**Affiliations:** 1 Cardiology Department, Ospedale Maggiore, 40100 Bologna, Italy; 2 Cardiology Department, Arnaud-De-Villeneuve Hospital, 34090 Montpellier, Francef-roubille@chu-montpellier.fr (F.R.)

**Keywords:** wearable cardioverter defibrillator, sudden cardiac death, acute cardiac care unit, intensive care unit, primary prevention, length of hospitalization, length of stay

## Abstract

The wearable cardioverter defibrillator (WCD) has been proven to be effective in preventing sudden cardiac death (SCD) in patients soon after acute myocardial infarction (AMI) and left ventricular ejection fraction (LVEF) ≤35%. The aim of this study was to assess whether a WCD may shorten the length of an initial hospital stay (total length, days in the intensive care unit (ICU) and in the acute cardiac care unit (ACCU)) among these patients. This was a single-centre, retrospective observational study of patients referred for the management of SCD risk post-AMI and LVEF ≤35%, in a tertiary care hospital. The clinical characteristics and length of index hospitalization of the group of patients discharged, with or without WCD, were compared. A propensity score analysis was performed, then weighted regression models were conducted. A total of 101 patients in the WCD group and 29 in the control group were enrolled in the analysis. In the weighted regression models, WCD significantly reduced the days spent in ACCU (*p* < 0.001). WCD patients had significantly fewer days spent in ACCU (5.5 ± 2.6 vs. 8.4 ± 12.8 days, *p* < 0.001) and shorter hospitalizations (10.2 ± 5.7 vs. 13.4 ± 17.6 days, *p* = 0.005), compared with the control group. It was concluded that the WCD appears to reduce the total length of hospitalization and lengths of stay in ACCU for patients post-AMI and with left ventricular dysfunction.

## 1. Introduction

The risk of sudden cardiac death (SCD) is increased after an acute myocardial infarction (AMI). A severe reduction of left ventricular ejection fraction (LVEF) is the strongest independent predictor of SCD soon thereafter [1]. Randomized clinical trials showed that implantable cardioverter-defibrillators (ICDs), in combination with optimal medical therapy (OMT), reduce the risk of SCD fourfold in patients who experience AMI with reduced LVEF [2,3]. However, randomized controlled trials did not provide empirical support for ICD implantation during the early-phase post-AMI period (particularly within 40 days) [4,5,6]. Furthermore, a significant proportion of patients improve their LVEF within the first three months. This is partly due to the slow up-titration of OMT in combination with cardiac remodeling during that time [7]. The wearable cardioverter defibrillator (WCD) can close the gap between hospital discharge and the best possible recovery obtained with medications.

The WCD (LifeVest^®^, ZOLL Medical Corporation, Pittsburgh, PA, USA) is a unique totally external medical device capable of detecting life-threatening arrhythmias and delivering the appropriate shocks for their termination [8,9].

Numerous prospective and retrospective studies have demonstrated the safety and effectiveness of the WCD [10,11,12]. The cost effectiveness of the WCD was analyzed in several publications in American and Italian settings [13,14,15,16]. Results of the studies indicated that the device was cost-effective, and one study found that the WCD was cost-saving, even when used after ICD explantation [17].

However, no studies to date have evaluated the direct impact of a WCD on the length of hospital stays among post-AMI patients.

Depending on the clinical presentation of patients with acute coronary syndrome (ACS), these patients can be managed in coronary intensive care units (ACCU) or an intensive care unit (ICU) [18,19]. Furthermore, in patients at high risk for SCD, the WCD is prescribed in the hospital prior to discharge.

The aim of our study was to assess whether a WCD could reduce total hospital stays, as well as the length of stay in ICU and ACCU, for post-AMI patients with severely reduced LVEF.

## 2. Materials and Methods

We conducted a single-centre, retrospective, observational study of consecutive post-AMI patients with LVEF ≤35%, over 18 years of age, admitted to the French University Hospital Arnaud de Villeneuve in Montpellier. Approval for the study was obtained from the institutional review board at Arnaud de Villeneuve Teaching Hospital in Montpellier, France (number IRB-MTP_2022_07_202201174).

Patients with the above-described inclusion criteria and additional WCD prescriptions, between June 2016 and March 2022, served as the study group. Patients discharged without a WCD (or ICD), from January to December 2022, formed the control group.

Patients who already had an ICD or cardiac resynchronization therapy defibrillator (CRT-D), were awaiting heart transplantation, had non-ischemic heart failure, LVEF >35% or were prescribed a WCD after ICD explantation, were excluded.

Components, functionality, and indications of the WCD are described in detail elsewhere [20,21].

For both study groups, the index event date began with the date of hospital admission for AMI. Baseline data included demographics, past medical history, and baseline medication. Electrocardiogram (ECG) and laboratory data were obtained from electronic medical records.

Echocardiographic parameters (such as left ventricular size and ejection fraction, right ventricular function, the presence of severe valvulopathy, as well as the presence of a left ventricular aneurysm or intraventricular thrombosis) were collected from the echocardiographic examination performed during the hospital stay and after coronary angiography (mean time of about five days post-AMI).

Data regarding the duration of hospitalization included total length of hospital stay, and days spent in ICU and ACCU.

For the WCD group, data on treatments were retrieved from the Zoll network database (LifeVest^®^ Zoll Medical Corp., Pittsburgh, PA, USA).

Each patient was followed to gather information about re-hospitalizations, ICD implantations, or death.

### Statistical Analysis

Continuous data were tested for normal distribution with the Kolmogorov–Smirnov test. Normally distributed continuous variables were presented as mean and standard deviations (SD). Continuous variables with skewed distributions were reported as medians and interquartile ranges (IQR). Categorical variables were summarized in terms of counts and percentages. Comparisons of continuous variables between groups were performed with Student’s *t*-test or the non-parametric Mann–Whitney test, as suitable. Categorical variables were compared with the Chi-square test.

To reduce the influences of potential selection bias, propensity score analyses were performed. In a first step, variables with an influence on WCD use were identified using a forward selection procedure. Variables with a potential influence were chosen a priori: age, gender, LVEF, ST-elevation ACS, multivessel disease, multivessel percutaneous coronary intervention, left ventricular aneurysm, left ventricular thrombosis and antiarrhythmic drugs use. Second, a logistic regression model adjusted for the selected variables was performed and inverse probability of treatment weighting was used to calculate weights for each patient. Finally, weighted regression models were conducted for total hospitalization, days in ICU and days in ACCU with WCD use as independent variables, respectively.

The following sensitivity analyses were performed: (1) weighted models were additionally adjusted for extra-corporeal membrane oxygenation (ECMO) use and heart transplantation (HTX); (2) variable selection, weighting and regression models were repeated excluding patients with ECMO or HTX; and (3) patients with longer hospital stays (more than 30 days) were excluded.

All *p* values < 0.05 were considered statistically significant. Data were analyzed using SAS (version 9.4).

## 3. Results

**Figure 1:**
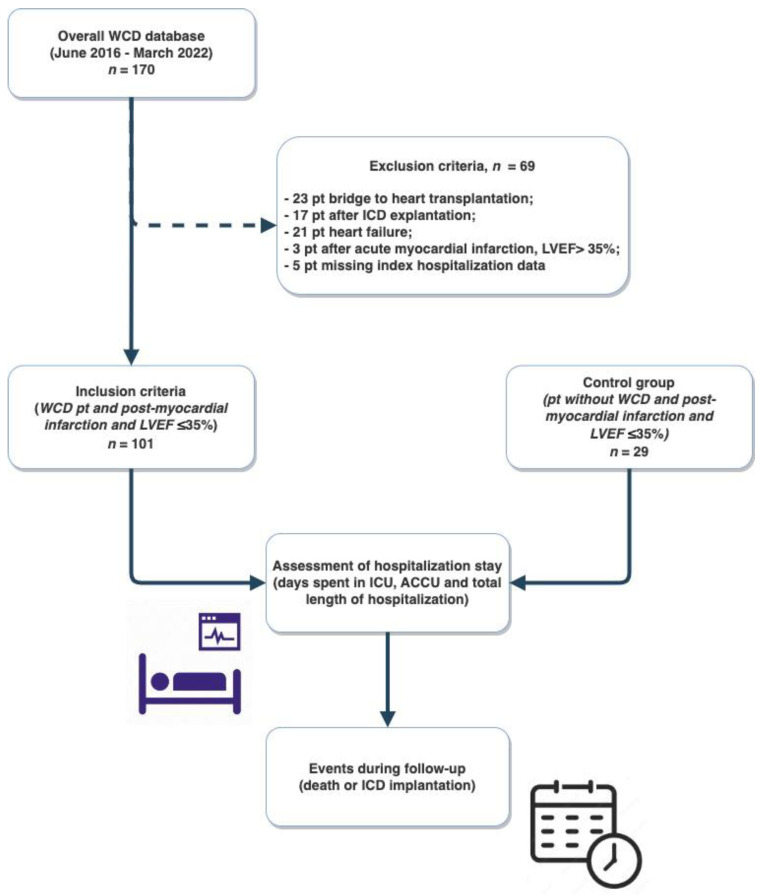
Study flow-chart. WCD, wearable cardioverter defibrillator; pt, patients; ICD, implantable cardioverter-defibrillator; LVEF, left ventricular ejection fraction; ICU, intensive care unit; ACCU, acute cardiac care unit.

One hundred and thirty consecutive post-AMI patients with LVEF ≤35% were included in the study: 101 patients were discharged with a WCD while 29 were not (Figure 1).

Mean age was 60.6 years (±10.9), 18.5% were female and 82.3% of patients had an ST-elevation ACS. All patients underwent coronary angiography after a mean time of 0 days (±1).

All patients were equipped with WCD during index hospitalization and previous to discharge, after a median time of 6.0 days (IQR 4.0–9.0) from the date of hospital admission. Baseline characteristics of the patients are depicted in Table 1. Clinical complications and the main resources used during the entire index hospitalization are shown in Table 2. 

Patients in the WCD group had significantly lower LVEF (28.2 ± 4.7 versus 32.1 ± 3.6; *p* < 0.001) and more cardiogenic shocks (23.8% versus 6.9%; *p* = 0.045).

ECMO was used with 11 patients (9 in the study group and 2 in the control group). In these patients, WCD was placed after weaning from ECMO (in seven patients) and earlier in two patients who subsequently had hemodynamic decompensation requiring circulatory support.

Three patients underwent HTX (two in the WCD group and one in the control group) in all cases after ECMO use.

The independent variables for WCD use were LVEF (*p* < 0.001) and left ventricular thrombosis (*p* = 0.034).

### 3.1. Hospitalization Times

In the context of management and treatment of ACS, 126 patients (96.9%) required ACCU care: 99 in the WCD group and 27 in the control group. Thirty-one patients (23.8%) required ICU care: 25 in the WCD group and 6 in the no-WCD group. Subsequently, all patients were transferred to the cardiology ward, from where they were discharged (except patients who died during hospitalization).

The different analyzes of hospitalization times are shown in Table 3 (a–c) and Supplementary Table S1.

In the general descriptive analysis, the mean hospital stay was 13.5 days (±13.0); mean days spent in ICU were 1.8 days (±5.1); and mean days in ACCU were 5.8 days (±3.7). The maximum number of days spent in hospital was higher in the WCD group compared to the control group (total hospital stay = 74 vs. 56 days, *p* = 0.196; ICU stay = 33 vs. 19 days, *p* = 0.557; ACCU stay = 28 vs. 17 days, *p* = 0.100); however, the number was without statistical significance.

The use of ECMO and HTX emerged as independent variables for a higher total length of stay (*p* < 0.001) and for time spent in ICU (*p* < 0.001). HTX, but not ECMO use, emerged as an independent variable for days spent in ACCU (*p* < 0.001 and *p* = 0.739, respectively).

In the weighted regression model (adjusted for ECMO use and HTX), compared with the no-WCD group, WCD significantly reduced days spent in ACCU (5.8 ± 3.9 vs. 8.9 ± 13.5 days, *p* < 0.001), but not the total hospital stay length (13.6 ± 14.6 vs. 17.2 ± 36.3 days, *p* = 0.143) or days in ICU (1.7 ± 5.6 vs. 2.9 ± 15.4 days, *p* = 0.251).

After excluding patients from the analysis with ECMO/HTX, WCD use resulted in a borderline significance for fewer total lengths of hospitalization (10.4 ± 7.2 vs. 12.4 ± 15.2 days, *p* = 0.066), compared with the no-WCD group. Again, the WCD reduced the days spent in ACCU (5.7 ± 3.5 vs. 9.1 ± 14.6 days, *p* < 0.001).

Finally, excluding patients with hospital stays >30 days (nine patients in the study group and one patient in the control group), resulted in significantly fewer total lengths of hospitalization (10.2 ± 5.7 vs. 13.4 ± 17.6 days, *p* = 0.005), as well as ACCU stays (5.5 ± 2.6 vs. 8.4 ± 12.8 days, *p* < 0.001) for patients with a WCD. Days in ICU were not significantly reduced (0.7 ± 2.3 vs. 1.2 ± 8.2, *p* = 0.356). In this latest analysis, it was found that patients with a WCD spent an average of 3.2 days less in the hospital than those without a WCD, and an average of 3.0 days less in the ACCU.

No patients experienced WCD shock during index hospitalization. All patients were discharged, except three who died during index hospitalization: two in the WCD group for non-cardiovascular causes (hemorrhagic stroke and septic shock, respectively), and one in the no-WCD group (for cardiogenic shock).

### 3.2. Follow-Up Period

Median WCD wear time from the patients was 34 days (IQR 18–59 days), while the maximum wear time was 136 days. The main reasons for discontinuing the WCD were improvement of LVEF in 43 patients (44.8%), and ICD implantation in 45 patients (46.9%). Five patients discontinued WCD use for lack of tolerance, and three patients died (3.1%).

Four patients experienced WCD shock and were subsequently hospitalized: of these, two patients received seven appropriate shocks, while another two patients received one inappropriate treatment each. In no cases did the inappropriate shock from the WCD result in a patient’s death. All patients with appropriate WCD shocks underwent subsequent ICD implantation. No further adverse events were reported.

After a mean follow-up period of 37.3 months (±25.3), 46 (49.5%) patients underwent ICD implantation in the WCD group (after a mean time of 72.3 ± 44.5 days). In the control group, 11 patients (39.3%) underwent ICD implantation (after a mean time of 69.6 ± 69.5 days), without statistically significant difference (*p* = 0.344).

During the same time period, one patient died after hospitalization in the control group, and three patients died in the WCD group. All deaths occurred after WCD discontinuation.

## 4. Discussion

Our study was the first to show that WCD shortens the hospital stays of post-AMI patients with ventricular dysfunction, especially in terms of days spent in ACCU.

Compared with the general population, patients who experience an AMI are four to six times more likely to suffer from an SCD. Arrhythmic risk is at its maximum in the first month and decreases exponentially over the first six months [1]. ICDs for the primary prevention of SCDs in the early phase after an AMI (<40 days) failed to reduce overall mortality due to an increase in non-SCD and other adverse events, such as infection, inappropriate shock and heart failure. In addition, some patients regained full cardiac function after the early-phase post-AMI period [2,3,4,5,6,7]. Thus, the WCD is an interesting device that could effectively fill this time-gap.

In a randomized trial, WCD significantly reduced total mortality but not arrhythmic mortality in the intention-to-treat analysis [11]. Further per protocol and as-treated analyses showed that WCD significantly reduced total mortality, as well as arrhythmic and non-arrhythmic death [12,15,16,17,18,19,22]. Importantly, several large observational studies unanimously showed improved WCD compliance compared to the randomized trial, suggesting that the results of the per protocol and as-treated analyses are closer to real-life clinical practice [23]. For this reason, international guidelines suggest considering the use of WCDs, among other indications, in selected patients after the early phase of AMI [24,25].

Despite several cost-effectiveness analyses on the use of the WCD, no study until now has directly analysed the implications of WCD use on the total duration of hospitalization, and lengths of stay in ICU and ACCU.

In weighted regression models, the WCD significantly reduced the days spent in ACCU and this remained stable even after adjusting for a greater burden of cardiovascular disease (*p* < 0.001). After excluding outliers with long hospitalizations, WCD use resulted in significantly fewer days spent in ACCU (*p* < 0.001) and shorter total length of hospitalization (*p* = 0.005), compared with control group.

Patients with WCDs spent an average 3.2 days less in the hospital than those without WCDs, and an average of 3.0 days less in the ACCU.

In no weighted regression models, the WCD resulted in shorter ICU lengths of stay, although not statistically significant. This finding is in line with real-world data, in which the WCD is placed shortly before discharge from high-monitoring cardiology departments (e.g., ACCU), but does not impact the length of stay in ICUs.

In our study, patients in the WCD group had a higher disease burden than those in the control group, and notably had significantly lower LVEF and worse renal function (e.g., peak creatinine level). Additionally, patients in the WCD group experienced significantly more frequent cardiogenic shocks. The propensity score analyses were mandatory to adjust for diverging baseline variables of the groups, in order to provide more reliable comparison between groups. In this perspective, non-significant differences in mortality and ICD implantation rates may indicate better patient management and outcomes with a WCD.

It comes as no surprise that LVEF emerged as the most important independent variable for the use of a WCD, even after excluding patients from the analysis with long hospitalizations and those who required ECMO or HTX. LVEF is the most important SCD-risk marker, and therefore part of the WCD indication [1,3].

About 40% of patients in both groups presented with a late STE-ACS (>12 h). This finding is in line with real-world data. Patients who might benefit from WCD and/or ICD often have large myocardial infarction, and late presentation might be the cause, resulting in a poor chance of revascularization [26]. In this group of patients, WCD often finds a bridge indication to ICD because of the high arrhythmic risk of these patients.

Overall, post-AMI patients often undergo lengthy hospitalizations. This is linked to the intrinsic instability of the cardiological condition. In other cases, the clinician perceives the risk of SCD as too high for hospital discharge. The WCD allows for these patients to be safely discharged, so that they can return to their home environment under protected conditions, without increasing the risk of SCD. WCD allows the patient to enter a “virtual clinic” where there is 24/7 continuous monitoring. In contrast, for patients who still require hospitalization or cardiac rehabilitation, WCD allows patients to be transferred to wards with a lower degree of monitoring in complete safety because they are protected from an SCD. WCD acceptance by patients is high, enabling them to conduct daily life activities without major restrictions. Therefore, the WCD can help to prevent unnecessary ICD implantation (almost half of the patients improved their LVEF until the end of WCD use), and thereby contribute to the improvement of quality of life as well as reduce healthcare costs. This efficacy is not only expressed in a direct reduction of healthcare costs but indirectly allows for a reduction in the complications correlated with prolonged hospital stays or unnecessary ICD implantation [27,28].

The length of stay in our study tended to be longer than those reported in the literature [29] for the same patient population. The retrospective setting of our study does not allow us to arrive at firm conclusions; however, some assumptions can be made. In fact, 11 patients required ECMO. Of these, three required subsequent cardiac transplantation, which undoubtedly lengthened the overall hospitalization times. To this should be added that one-third of the patients in both groups underwent multivessel percutaneous coronary intervention, which was not always performed during the first session, and certainly lengthened the hospital stay.

The rate of appropriate WCD shocks in our study was 1.98%, and the same was reported for inappropriate shocks. This indicates that use of the WCD helped to prevent two serious cardiovascular events. The results are in line with those published in the literature [10,30]. Therefore, the WCD is a useful and feasible device for the prevention of SCD in post-AMI patients with severe left ventricular dysfunction. The WCD has an excellent cost-effectiveness and is able to reduce the duration of hospitalizations, especially stays in ACCU.

### Limitations

Our study has several limitations that warrant consideration. First, this was a single-centre analysis with a retrospective design performed in a non-randomized manner. This poses some inherent limitations, including selection bias and unknown confounding factors that could not be controlled. Second, there was a limited number of patients included with some important differences between groups. Thus, we tried to reduce the impact of selection bias by constructing different sensitivity analysis models assessing the effects of various measurable and unmeasurable confounders. Third, our results may not be generalizable to hospitals with different organizational levels, especially for patients with lower disease complexity. Fourth, our study did not include analyses of some laboratory and echocardiographic parameters over time. In addition, an analysis of hemodynamic parameters or cognitive function, which might have indirectly influenced the choice to place WCD, was not included. Subsequent analyses may focus specifically on the parameters listed above. Finally, it is possible to imagine a direct reduction in healthcare costs with the use of WCDs; however, our analysis is currently unable to answer this question. A sub-analysis of the costs of WCDs versus hospitalization is still ongoing.

## 5. Conclusions

The WCD was effective for the outpatient management of patients at a high risk of an SCD following a myocardial infarction. Our data support that WCD may allow for earlier discharges, resulting in fewer days spent in ACCU and lower hospital lengths of stay. This is important to consider within the context of modern hospital organizations, and in view of the better allocation of economic resources. Confirmation by a prospective randomized clinical trial is as necessary as ever.

## Figures and Tables

**Table 1:** Baseline clinical characteristics of the patients.

	Total *n* = 130	Life Vest *n* = 101	Control *n* = 29	*p* Value
Age (years)—mean (±SD)	60.6 ± 10.9	60.3 ± 10.4	61.7 ± 12.8	0.532
Male sex—n. (%)	106 (81.5)	81 (80.2)	25 (86.2)	0.462
Past Medical history—no. (%)				
Hypertension	53 (40.8)	40 (39.6)	13 (44.8)	0.614
Dyslipidemia	35 (26.9)	26 (25.7)	9 (31.0)	0.571
Type 2 diabetes	23 (17.7)	16 (15.8)	7 (24.1)	0.302
Smoking history	87 (66.9)	65 (64.4)	22 (75.9)	0.246
Stroke/TIA	9 (6.9)	9 (8.9)	0 (0.0)	0.096
COPD	9 (6.9)	8 (7.9)	1 (3.4)	0.403
Chronic renal failure	7 (5.4)	7 (6.9)	0 (0.0)	0.145
Previous CAD	18 (13.8)	13 (12.9)	5 (17.2)	0.548
Previous PCI	11 (8.5)	7 (6.9)	4 (13.8)	0.241
Previous CABG	2 (1.5)	2 (2.0)	0 (0.0)	0.445
Peripheral vascular disease	4 (3.1)	3 (3.0)	1 (3.4)	0.896
Atrial fibrillation	9 (6.9)	7 (6.9)	2 (6.9)	0.995
Index event—n. (%)				
ACS type				
STE-ACS early (≤12 h)	55 (42.3)	41 (40.6)	14 (48.3)	0.239
STE-ACS late (>12 h)	52 (40.0)	40 (39.6)	12 (41.4)	0.863
NSTE-ACS	23 (17.7)	19 (19.8)	3 (10.3)	0.402
Multivessel disease	90 (69.2)	72 (71.3)	18 (62.1)	0.343
PCI	122 (93.8)	94 (93.1)	28 (96.6)	0.492
Multivessel PCI	47 (36.2)	37 (36.3)	10 (34.5)	0.832
In-hospital echocardiographic evaluation				
LVEF %—mean (±SD)	29.0 ± 4.7	28.1 ± 4.7	32.1 ± 3.6	<0.001
LVEDD (mm)—mean (±SD)	55.8 ± 7.4	56.1 ± 7.4	54.8 ± 7.7	0.560
LVEDV (ml)—mean (±SD)	165.3 ± 57.4	172.5 ± 60.6	138.3 ± 32.7	0.013
Right ventricular dysfunction—n. (%)	10 (7.9)	8 (8.2)	2 (7.1)	0.860
Severe valvulopathy—n (%)	7 (5.4)	7 (7.0)	0 (0.0)	0.143
Aneurysm—n. (%)	12 (9.4)	10 (10.2)	2 (6.9)	0.593
Left ventricular thrombosis—n. (%)	25 (19.2)	23 (22.8)	2 (6.9)	0.056
In-hospital electrocardiographic evaluation at presentation				
Sinus rhythm—n. (%)	122 (93.8)	95 (94.1)	27 (93.1)	0.850
Duration of the QRS interval (msec)—mean (±SD)	100.5 ± 20.0	102.4 ± 20.4	94.0 ± 17.5	0.055
QTc duration (msec)—mean (±SD)	429.2 ± 36.0	432.2 ± 33.9	418.3 ± 41.7	0.076
Bundle branch block—n. (%)	22 (17.5)	19 (19.2)	3 (11.1)	0.327
In-hospital laboratory evaluation at presentation				
Anemia (Hb < 11 g/dL)—n. (%)	7 (5.4)	7 (6.9)	0 (0.0)	0.348
White blood cells count (10^9^/L), mean (±SD)	13.6 ± 4.9	13.4 ± 4.8	14.3 ± 5.3	0.338
Platelet (10^9^/L)—mean (±SD)	260.9 ± 96.4	258.7 ± 89.7	268.8 ± 118.2	0.673
Creatinine (µmol/L)—mean (±SD)	91.0 ± 35.0	92.0 ± 37.5	87.9 ± 23.9	0.485
Laboratory evaluation during hospitalization				
Creatinine peak (µmol/L)—mean (±SD)	117.7 ± 55.1	121.5 ± 60.5	104.1 ± 24.4	0.025
TnT hs peak (ng/L)—mean (±SD)	8516.2 ± 7503.6	8517.5 ± 7881.3	8511.6 ± 6128.8	0.997
NT-proBNP peak (ng/L)—median (IQR)	3920.0 (1808.5–7073.5)	3999.5 (1968.0–7285.3)	1537.0 (3224.0–5149.5)	0.169

SD, standard deviation; TIA, transient ischemic attack; COPD, chronic obstructive pulmonary disease; CAD, coronary artery disease; PCI, percutaneous coronary intervention; CABG, coronary artery bypass graft surgery; ACS, acute coronary syndrome; STE, ST-elevation; NSTE, non-ST-elevation; LVEF, left ventricular ejection fraction; LVEDD, left ventricular end-diastolic diameter; LVEDV, left ventricular end-diastolic volume; TnT, troponin T.

**Table 2:** Clinical complications and the main resources used during the entire index hospitalization.

	Total *n* = 130	Life Vest *n* = 101	Control *n* = 29	*p* Value
CABG	3 (2.3)	3 (3.0)	0 (0.0)	0.348
Impella	8 (6.2)	7 (6.9)	1 (3.4)	0.492
ECMO	11 (8.5)	9 (8.9)	2 (6.9)	0.731
Heart transplant	3 (2.3)	2 (2.0)	1 (3.4)	0.643
Dialysis	2 (1.5)	2 (2.0)	0 (0.0)	0.445
Non-invasive ventilation (C-PAP or BiPAP)	7 (5.4)	7 (6.9)	0 (0.0)	0.145
Mechanical ventilation	15 (11.5)	13 (12.9)	2 (6.9)	0.374
Temporary pacemaker	3 (2.3)	3 (3.0)	0 (0.0)	0.347
RBC transfusion	11 (8.5)	10 (9.9)	1 (3.4)	0.271
Inotropic drugs	19 (14.6)	17 (16.8)	2 (6.9)	0.182
Vasopressor drugs	21 (16.2)	18 (17.8)	3 (10.3)	0.335
Complications during hospitalization—n. (%)				
Stroke	3 (2.3)	3 (3.0)	0 (0.0)	0.348
Cardiogenic shock	26 (20.0)	24 (23.8)	2 (6.9)	0.045
Pulmonary embolism	0 (0.0)	0 (0.0)	0 (0.0)	-
Sepsis	12 (9.2)	10 (9.9)	2 (6.9)	0.622
Acute kidney failure ^a^	62 (47.7)	52 (51.5)	10 (34.5)	0.106
Bradyarrhythmias	2 (1.5)	2 (2.0)	0 (0.0)	0.445
Ventricular tachyarrhythmias ^b^	68 (52.3)	55 (54.5)	13 (44.8)	0.360
Therapy at discharge—n. (%)				
Angiotensin-converting enzyme inhibitors (ACEi) or angiotensin II receptor blockers (ARB)	114 (87.7)	89 (88.1)	25 (86.2)	0.659
Angiotensin Receptor-Neprilysin Inhibitor	2 (1.6)	2 (2.0)	0 (0.0)	0.453
Beta-blockers	125 (96.9)	99 (98.0)	26 (92.9)	0.163
Mineralocorticoid Receptor Antagonists	106 (82.2)	87 (86.1)	19 (67.9)	0.025
Sodium-glucose cotransporter 2 inhibitors (SGLT2i)	26 (20.2)	24 (23.8)	2 (7.1)	0.052
Ivabradine	9 (7.0)	7 (6.9)	2 (7.1)	0.969
Diuretics	65 (50.4)	56 (55.4)	9 (32.1)	0.029
Statin	118 (91.5)	92 (91.1)	26 (92.9)	0.767
Acetylsalicylic acid	120 (93.0)	93 (92.1)	27 (96.4)	0.424
P2Y12 receptor blockers	119 (92.2)	94 (93.1)	25 (89.3)	0.508
Novel Oral Anticoagulants/Warfarin	40 (31.0)	33 (32.7)	7 (25.0)	0.437
Amiodarone	13 (10.1)	11 (10.9)	2 (7.1)	0.559

^a^ Defined as an increase in creatinine values greater than or equal to 25% compared to the initial ones. ^b^ Including ventricular fibrillation, sustained ventricular tachycardia, non-sustained ventricular tachycardia. CABG, coronary artery bypass graft surgery; ECMO, extracorporeal membrane oxygenation; C-PAP, continuous positive airway pressure; Bi-PAP, bi-level positive airway pressure; RBC, red blood cell.

**Table 3:** (**a**)— Base propensity score model for hospitalization times. (**b**)— Propensity score model for hospitalization times without ECMO and HTX. (**c**)— Propensity score model for hospitalization times without patients with long (>30 days) hospital stay.

(**a**)					
**Base Model**	**Total** ***n* = 130** **mean (±SD)**	**Life Vest** ***n* = 101** **mean (±SD)**	**Control** ***n* = 29** **mean (±SD)**	**Estimate Effect [95%-CI]**	***p* Value**
Total hospital length (days)	15.7 ± 21.4	13.6 ± 14.6	17.2 ± 36.3	−3.5 [−8.4; 1.2]	0.143
Days in ICU (days)	2.4 ± 8.8	1.7 ± 5.6	2.9 ± 15.4	−1.1 [−3.1; 0.8]	0.251
Days in ACCU (days)	7.6 ± 7.5	5.8 ± 3.9	8.9 ± 13.5	−3.1 [−4.7; −1.4]	<0.001
Weighted number of days adjusted for extra-corporeal membrane oxygenation (ECMO) use and heart transplantation (HTX). SD, standard deviation; ICU, intensive care unit; ACCU, acute cardiac care unit.					
(**b**)					
**Model without ECMO, HTX**	**Total** ***n* = 119** **mean (±SD)**	**Life Vest** ***n* = 92** **mean (±SD)**	**Control** ***n* = 27** **mean (±SD)**	**Estimate Effect [95%-CI]**	***p* Value**
Total hospital length (days)	11.8 ± 9.7	10.4 ± 7.2	12.4 ± 15.2	−2.0 [−4.1; 0.1]	0.066
Days in ICU (days)	0.3 ± 1.8	0.6 ± 1.7	0.2 ± 1.8	0.3 [0.0; 0.7]	0.079
Days in ACCU (days)	7.9 ± 8.0	5.7 ± 3.5	9.1 ± 14.6	−3.4 [−5.1; −1.7]	<0.001
Weighted number of days after exclusion of patients with extra-corporeal membrane oxygenation (ECMO) and heart transplantation (HTX). SD, standard deviation; ICU, intensive care unit; ACCU, acute cardiac care unit.					
(**c**)					
**Model without hospital stays >30 days**	**Total** ***n* = 120** **mean (±SD)**	**Life Vest** ***n* = 92** **mean (±SD)**	**Control** ***n* = 28** **mean (±SD)**	**Estimate Effect [95%-CI]**	***p* Value**
Total hospital length (days)	12.2 ± 10.1	10.2 ± 5.7	13.4 ± 17.6	−3.2 [−5.4; −1.0]	0.005
Days in ICU (days)	1.0 ± 4.4	0.7 ± 2.3	1.2 ± 8.2	−0.5 [−1.5; 0.5]	0.356
Days in ACCU (days)	7.3 ± 6.9	5.5 ± 2.6	8.4 ± 12.8	−3.0 [−4.4; −1.5]	<0.001
Weighted number of days after exclusion of patients with hospital stays > 30 days. SD, standard deviation; ICU, intensive care unit; ACCU, acute cardiac care unit.					

## Data Availability

The data presented in this study are available on request from the corresponding author. The data are not publicly available due to restrictions eg privacy or ethical.

## Supplementary Materials

The following supporting information can be downloaded at: https://www.mdpi.com/article/10.3390/jcm12154884/s1, Table S1: Hospitalization times, descriptive analysis.

## Abbreviations

ACCU	Acute cardiac care unit
ACS	Acute coronary syndrome
AMI	Acute myocardial infarction
CRT-D	Cardiac resynchronization therapy defibrillator
ECG	Electrocardiogram
ECMO	Extra-corporeal membrane oxygenation
HTX	Heart transplantation
ICD	Implantable cardioverter-defibrillator
ICU	Intensive care unit
IQR	Interquartile ranges
LVEF	Left ventricular ejection fraction
OMT	Optimal medical therapy
SCD	Sudden cardiac death
SD	Standard deviation
WCD	Wearable cardioverter defibrillator

## References

[1] Solomon S.D., Zelenkofske S., McMurray J.J., Finn P.V., Velazquez E., Ertl G., Harsanyi A., Rouleau J.L., Maggioni A., Kober L. (2005). Sudden Death in Patients with Myocardial Infarction and Left Ventricular Dysfunction, Heart Failure, or Both. N. Engl. J. Med..

[2] Buxton A.E., Lee K.L., Fisher J.D., Josephson M.E., Prystowsky E.N., Hafley G. (1999). A Randomized Study of the Prevention of Sudden Death in Patients with Coronary Artery Disease. N. Engl. J. Med..

[3] Elayi C.S., Charnigo R.J., Heron P.M., Lee B.K., Olgin J.E. (2017). Primary Prevention of Sudden Cardiac Death Early Post-Myocardial Infarction. Circ. Arrhythmia Electrophysiol..

[4] Moss A.J., Zareba W., Hall W.J., Klein H., Wilber D.J., Cannom D.S., Daubert J.P., Higgins S.L., Brown M.W., Andrews M.L. (2002). Prophylactic implantation of a defibrillator in patients with myocardial infarction and reduced ejection fraction. N. Engl. J. Med..

[5] Steinbeck G., Andresen D., Seidl K., Brachmann J., Hoffmann E., Wojciechowski D., Kornacewicz-Jach Z., Sredniawa B., Lupkovics G., Hofgärtner F. (2009). Defibrillator Implantation Early after Myocardial Infarction. N. Engl. J. Med..

[6] Hohnloser S.H., Kuck K.H., Dorian P., Roberts R.S., Hampton J.R., Hatala R., Fain E., Gent M., Connolly S.J. (2004). Prophylactic Use of an Implantable Cardioverter–Defibrillator after Acute Myocardial Infarction. N. Engl. J. Med..

[7] Solomon S.D., Glynn R.J., Greaves S., Ajani U., Rouleau J.-L., Menapace F., Arnold J.M.O., Hennekens C., Pfeffer M.A. (2001). Recovery of ventricular function after myocardial infarction in the reperfusion era: The healing and early afterload reducing therapy study. Ann. Intern. Med..

[8] Chieng D., Paul V., Denman R. (2018). Current Device Therapies for Sudden Cardiac Death Prevention—The ICD, Subcutaneous ICD and Wearable ICD. Heart Lung Circ..

[9] Piccini J.P., Allen L.A., Kudenchuk P.J., Page R.L., Patel M.R., Turakhia M.P. (2016). Wearable Cardioverter-Defibrillator Therapy for the Prevention of Sudden Cardiac Death. Circulation.

[10] Feldman A.M., Klein H., Tchou P., Murali S., Hall W.J., Mancini D., Boehmer J., Harvey M., Heilman M.S., Szymkiewicz S.J. (2004). Use of a Wearable Defibrillator in Terminating Tachyarrhythmias in Patients at High Risk for Sudden Death: Results of WEARIT/BIROAD. Pacing Clin. Electrophysiol..

[11] Olgin J.E., Pletcher M.J., Vittinghoff E., Wranicz J., Malik R., Morin D.P., Zweibel S., Buxton A.E., Elayi C.S., Chung E.H. (2018). Wearable Cardioverter–Defibrillator after Myocardial Infarction. N. Engl. J. Med..

[12] Olgin J.E., Lee B.K., Vittinghoff E., Morin D.P., Zweibel S., Rashba E., Chung E.H., Borggrefe M., Hulley S., Lin F. (2020). Impact of wearable cardioverter-defibrillator compliance on outcomes in the VEST trial: As-treated and per-protocol analyses. J. Cardiovasc. Electrophysiol..

[13] Healy C.A., Carrillo R.G. (2015). Wearable cardioverter-defibrillator for prevention of sudden cardiac death after infected implantable cardioverter-defibrillator removal: A cost-effectiveness evaluation. Heart Rhythm..

[14] Sanders G.D., Owens D.K., Hlatky M.A. (2015). Potential Cost-effectiveness of Wearable Cardioverter-Defibrillator Early Post Myocardial Infarction. J. Innov. Card. Rhythm. Manag..

[15] Botto G.L., Mantovani L.G., Cortesi P.A., De Ponti R., D’Onofrio A., Biffi M., Capucci A., Casu G., Notarstefano P., Scaglione M. (2022). The value of wearable cardioverter defibrillator in adult patients with recent myocardial infarction: Economic and clinical implications from a health technology assessment perspective. Int. J. Cardiol..

[16] Clark M.A., Szymkiewicz S., Volosin K., Kabulski G.M., Northup A., Wiggins B.S. (2019). Mortality and Costs Associated with Wearable Cardioverter-defibrillators after Acute Myocardial Infarction: A Retrospective Cohort Analysis of Medicare Claims Data. J. Innov. Card. Rhythm Manag..

[17] Boriani G., Mantovani L.G., Cortesi P.A., De Ponti R., D’Onofrio A., Arena G., Curnis A., Forleo G., Guerra F., Porcu M. (2021). Cost-minimization analysis of a wearable cardioverter defibrillator in adult patients undergoing ICD explant procedures: Clinical and economic implications. Clin. Cardiol..

[18] Gage A., Higgins A., Lee R. (2022). Cardiac Critical Care: The Evolution of a Novel Subspecialty. Methodist DeBakey Cardiovasc. J..

[19] Chen R., Strait K.M., Dharmarajan K., Li S.-X., Ranasinghe I., Martin J., Fazel R., Masoudi F.A., Cooke C.R., Nallamothu B.K. (2015). Hospital variation in admission to intensive care units for patients with acute myocardial infarction. Am. Heart J..

[20] Reek S., Burri H., Roberts P.R., Perings C., Epstein A.E., Klein H.U., Lip G., Gorenek B., Sticherling C., Fauchier L. (2016). The wearable cardioverter-defibrillator: Current technology and evolving indications. Europace.

[21] Klein H.U., Goldenberg I., Moss A.J. (2013). Risk stratification for implantable cardioverter defibrillator therapy: The role of the wearable cardioverter-defibrillator. Eur. Heart J..

[22] Nguyen E., Weeda E., Kohn C., D’Souza B., Russo A., Noreika S., Coleman C. (2018). Wearable Cardioverter-defibrillators for the Prevention of Sudden Cardiac Death: A Meta-analysis. J. Innov. Card. Rhythm. Manag..

[23] Wäßnig N.K., Günther M., Quick S., Pfluecke C., Rottstädt F., Szymkiewicz S.J., Ringquist S., Strasser R.H., Speiser U. (2016). Experience with the Wearable Cardioverter-Defibrillator in Patients at High Risk for Sudden Cardiac Death. Circulation.

[24] Zeppenfeld K., Tfelt-Hansen J., de Riva M., Winkel B.G., Behr E.R., Blom A.N., Charron P., Corrado D., Dagres N., de Chillou C. (2022). 2022 ESC Guidelines for the management of patients with ventricular arrhythmias and the prevention of sudden cardiac death. Eur. Heart J..

[25] Bodin A., Bisson A., Fauchier L. (2021). When is a wearable defibrillator indicated?. Expert Rev. Med Devices.

[26] Cho K.H., Han X., Ahn J.H., Hyun D.Y., Kim M.C., Sim D.S., Hong Y.J., Kim J.H., Ahn Y., Hwang J.Y. (2021). Long-Term Outcomes of Patients with Late Presentation of ST-Segment Elevation Myocardial Infarction. J. Am. Coll. Cardiol..

[27] Sud M., Yu B., Wijeysundera H.C., Austin P.C., Ko D.T., Braga J., Cram P., Spertus J.A., Domanski M., Lee D.S. (2017). Associations Between Short or Long Length of Stay and 30-Day Readmission and Mortality in Hospitalized Patients With Heart Failure. JACC Heart Fail..

[28] Hoogervorst-Schilp J., Langelaan M., Spreeuwenberg P., De Bruijne M.C., Wagner C. (2015). Excess length of stay and economic consequences of adverse events in Dutch hospital patients. BMC Health Serv. Res..

[29] Toušek P., Bauer D., Neuberg M., Nováčková M., Mašek P., Tu˚ma P., Kočka V., Moťovská Z., Widimský P. (2022). Patient characteristics, treatment strategy, outcomes, and hospital costs of acute coronary syndrome: 3 years of data from a large high-volume centre in Central Europe. Eur. Heart J. Suppl..

[30] Chung M.K., Szymkiewicz S.J., Shao M., Zishiri E., Niebauer M.J., Lindsay B.D., Tchou P.J. (2010). Aggregate National Experience with the Wearable Cardioverter-Defibrillator: Event Rates, Compliance, and Survival. J. Am. Coll. Cardiol..

