# Supplementary material for: Wearable Cardioverter Defibrillator Shortens the Lengths of Stay in Patients with Left Ventricular Dysfunction after Myocardial Infarction: A Single-Centre Real-World Experience
Source: J Clin Med. 2023 Jul 25;12(15):4884. doi: 10.3390/jcm12154884 (PMC10419391; doi:10.3390/jcm12154884)
Supplement: Supplementary file 1 [file jcm-12-04884-s001.zip › jcm-2472542-supplementary.pdf]

*Supplementary Table 1 - **Hospitalization times, descriptive analysis**. SD, standard deviation; ICU, intensive care unit; ACCU, acute cardiac care unit.*

	**Total** *n* **= 130**	**Life Vest** *n* **= 101**	**Control** *n* **= 29**	***p value***
Total hospital length (days) – mean (± SD)	13.5 ± 13.0	14.3 ± 13.6	10.8 ± 9.9	0.196
Hospitalization in ICU – n. (%)	31 (23.8)	25 (24.8)	6 (20.7)	0.806
Days in ICU (days) – mean (± SD)	1.8 ± 5.1	1.9 ± 5.3	1.3 ± 4.0	0.557
Hospitalization in ACCU – n. (%)	126 (96.9)	99 (98.0)	27 (93.1)	0.215
Days in ACCU (days) – mean (± SD)	5.8 ± 3.7	6.1 ± 3.7	4.8 ± 3.6	0.100
